# The use of mobile phone applications to enhance personal safety from interpersonal violence – an overview of available smartphone applications in the United Kingdom

**DOI:** 10.1186/s12889-022-13551-9

**Published:** 2022-06-09

**Authors:** Kat Ford, Mark A. Bellis, Natasha Judd, Nel Griffith, Karen Hughes

**Affiliations:** 1 Public Health Collaborating Unit, School of Medical and Health Sciences, College of Human Sciences, Bangor University, Wrexham, LL13 7YP UK; 2 World Health Organization Collaborating Centre on Investment for Health and Well-being, Policy and International Health, Public Health Wales, Wrexham, LL13 7YP UK

**Keywords:** Violence prevention, Interpersonal violence, Personal safety, Mobile technology, Mobile applications, Apps, Smartphones

## Abstract

**Background:**

Interpersonal violence has devastating implications for individuals, families, and communities across the globe, placing a significant burden on health, justice, and social welfare systems. Smartphone technology may provide a platform for violence prevention interventions. However, evidence on the availability and user experience of smartphone applications aimed to prevent violence is underexplored.

**Methods:**

Systematic searches of available smartphone applications marketed for personal safety and violence prevention on the Apple Store (IOS) and Google Play (Android) in the United Kingdom were run in May 2021. Relevant applications were downloaded, with data on user reviews and ratings extracted. Included applications were categorised according to their features and functions. Online user reviews were rated according to their sentiment (positive, negative, neutral) and thematically analysed.

**Results:**

Of 503 applications, 86 apps met review criteria. Only 52 (61%) apps offered full functionality free of charge. Over half (52%) of apps were targeted towards the general population, with 16% targeting women and 13% targeting families. App functionality varied with 22% providing an alarm, 71% sending alerts to pre-designated contacts, 34% providing evidence capture and 26% offering educational information. Overall, 71% of applications had a user rating of four or above. For 61 apps a total of 3,820 user reviews were extracted. Over half (52.4%) of reviews were rated as having a positive sentiment, with 8.8% neutral and 38.8% negative. Key themes across user reviews included positive consequences of app use, technical and usage issues including app reliability, dissatisfaction with the financial cost of some app features and personal data and ethical issues.

**Conclusions:**

Reviews suggest that users find apps for personal safety and violence prevention useful. However, individuals also report them being unreliable, not working as described and having features that others may exploit. Findings have implications for the development of policy on apps to improve personal safety, especially given recent national policy (e.g. UK) discussions about their utility. Without the regulation or accreditation of such technology for quality assurance and reliability, emphasis needs to be placed on ensuring user safety; otherwise vulnerable individuals may continue to place reliance on untested technology in potentially dangerous circumstances.

**Supplementary Information:**

The online version contains supplementary material available at 10.1186/s12889-022-13551-9.

## Background

Interpersonal violence is a leading cause of physical and psychological harm and early mortality [1, 2]. However, the impacts of interpersonal violence are widespread and not limited to the victim [3–6]. At a societal level, violence poses a significant economic burden via healthcare provision, loss of productivity, and criminal justice involvement [7]. As such, in 2015–16 in England and Wales, the total estimated cost of violence was £47.1 billion [8]. Consequently, violence prevention is a key feature of government legislation and policy, and a public health approach to violence prevention is progressively sought [9].

Research has explored how technology may stimulate violence, harassment, or abuse, for example, through violent video-game use [10–12]. However, technology may also provide a platform for violence prevention intervention strategies. Smartphone technology is increasingly targeted as a platform or resource for improving personal safety. Smartphones allow users access to advanced communication, information sharing and geolocation systems, and their possession has been linked to an increase in user perceptions of safety [13, 14]. There has also been a global escalation in smartphone ownership [15]. In 2020, 82% of adults (aged 16 years and over) in the United Kingdom (UK) were estimated to own a smartphone [16], up from 17% in 2008 [17]. Consequently, recent years have witnessed an increase in the number of smartphone applications, referred to here as ‘apps’, marketed as increasing personal safety by reducing an individual’s vulnerability to violence [18–21]. User demand for such apps has also increased; two thirds (62.9%) of participants in a study exploring mobile phone app preferences stated that they would consider downloading a personal safety app [22]. Furthermore, national policymakers (e.g. UK) are also starting to consider the potential utility of apps as a method to protect individuals, particularly women, from violence [23]. For individuals experiencing domestic abuse, the UK Government currently recommend the use of an app, which provides information on available support and how to recognise and respond to abuse [24]. Furthermore, as part of schemes to tackle violence against women and girls, in 2021, the UK Home Office provided funding to a smartphone app which monitors users’ locations and allows them to report unsafe areas [25]. However, the Home Office was criticised by many charities and organisations for their support of the app for its failure to tackle the root causes of violence against women and girls [26]. Furthermore, despite some pilot testing of the app by UK Police [25], no formal evaluation of the effectiveness of such technology has been undertaken.

Several smartphone features have the potential to enable users to avoid or mitigate violent situations, including GPS (Global Positioning System) tracking, remote-activated calling, and real-time user information. Other common facilities, such as camera and audio recording, may also support the evidence capture of violence [27]. Globally, high levels of violence are unreported and consequently unaddressed—in England and Wales, it is estimated that only 38% of violent crime is reported to the police [28]. Apps that capture accurate and detailed evidence have the potential to assist reports of violence. Furthermore, apps that provide tools and resources for violence prevention could potentially empower users to take control against violence in their own communities (actions which are recognised to be critical in the reduction of interpersonal violence [2]) and help influence social norms against violence (an established risk factor for all types of interpersonal violence [29]). Despite considerable commercial activity in the development of apps marketed to improve personal safety, little is known about the current availability of such apps, what features they contain and how they are being used. Such understanding might enable further knowledge on the potential of apps as either a violence reporting mechanism or a violence prevention intervention/strategy.

To date, research on smartphone apps marketed for violence prevention has predominately focused on intimate partner violence, sexual violence, or violence against women (VAW) more broadly [19, 30–35]. Findings of such work indicate that apps can assist users in accessing support and resources to increase their personal safety. A content analysis of the role of personal safety apps in reducing women’s vulnerability to public stranger violence in Australia found that, despite reducing a user’s fear of crime, apps had little usefulness in reducing victimisation [18]. A systematic review of apps in Europe and Central Asia to address VAW identified 43 apps, the majority of which were targeted for use in emergency situations, or provided education [21]. However, neither bodies of work included an examination of app user ratings or reviews. It is important that the app user experience is understood to enable a full exploration of the potential utility of such apps in preventing violence [36]. Such understanding is particularly important as research has highlighted concerns for the potential misuse of apps which offer location tracking by domestic abuse perpetrators, thus increasing risks to app users [37], and because apps can be developed and marketed without accreditation or regulation.

## Methods

This manuscript aims to describe the nature of freely available smartphone apps in the UK marketed to increase personal safety and prevent interpersonal violence. The review of available apps includes an assessment of app features and user experience and satisfaction with such apps.

### App identification

An electronic search of UK smartphone apps was conducted. The Apple Store (IOS) and Google Play (Android) were searched on 25^th^/26^th^ May 2021 using the terms “personal safety” and “violence prevention”. No restrictions were applied to the searches. Five hundred and three apps were retrieved across the searches (327 Android, 176 IOS). To account for software variation, apps available on both operating systems (*n* = 18; 36/503) were reviewed for each system independently. Information on retrieved apps were manually extracted into Microsoft Excel including name, store category, description and user rating (a numeric rating given to apps by users on a scale of 1–5 stars).

Apps were screened for inclusion by two reviewers using the following criteria: (1) the app description was in English; (2) stated a purpose to enhance personal safety or prevent violence; (3) offered services/features related to protection from, or prevention of, interpersonal violence; and (4) was freely available for download and use in the UK. At this stage, 332 apps were excluded because they were: unavailable for download in the UK or not in English language (*n* = 54); required corporate enterprise/university membership (*n* = 44); related to forms of safety distinct from interpersonal violence (e.g. online data/virus protection, *n* = 201); or had an associated cost (i.e. required a subscription or specific technology/device for use, *n* = 33). Included apps (*n* = 171) were then downloaded. At this stage, 85 apps were excluded because: after more than three attempts, the app did not function in the ways that it was marketed or described by the app developer (e.g. personal safety functions did not work; *n* = 45); the app did not work in the UK (*n* = 10); the app required payment on use (*n* = 16); or the app met other exclusion criteria (*n* = 14). Apps that offered a free functionality, with the additional availability of a paid-premium service, were included using the basic app functionality. In total, 86 apps were retained for analysis (see Table 1), for which data were extracted on: paid premium details (i.e. any information on cost or subscription), target app population, access requirements (e.g., app requiring access to contacts, microphone) and functions. Available English language user reviews and overall user ratings were extracted for a three-year period (26^th^ May 2018 – 26^th^ May 2021).

**Table 1:** Included apps, search term identifying app and platform app extracted from^a^

App name	Android	IOS
Aloha personal safety	PS	PS
Anjel		PS
Auggie personal safety		PS
Baxta—Personal Safety & Family Locator & Tracker	PS	
Bbguarder		PS
Beacon Safety	PS	
BEAWARE—Personal Safety App	PS	
bSafe—Never Walk Alone	PS	PS
Call For Help—Emergency SOS	PS	
CaringApp—Seniors and Caregivers Safety App	PS	
Chilla: Women safety app with scream detection	PS	
Demo Help—Personal Safety App	PS	
DocuSAFE Evidence Collection (Early Access)	VP	
Domestic Violence Prevention		VP
Emergency SOS Safety Alert Message	PS	
Eyes—Personal Safety & Streamlined Communication		PS
Feel Safe—Personal Safety		PS
Find my kids: Child GPS watch app & phone tracker	PS	
GeoLocator — Family Tracker + Baby Monitor Online	PS	
GetHomeSafe—Personal Safety	PS	PS
GruupUp—My safety		PS
Guardians—Personal & Family Safety	PS	
Guardians from Truecaller		PS
Heroes nearby		PS
Hollie Guard—Personal Safety App	PS	PS
I'M OK—Personal Safety App	PS	PS
iHELP Personal & Family Safety	PS	
InSec (Intelligent Security)—Personal Safety App	PS	
iOkay Personal Safety		PS
Jamie Kimble foundation		VP
KASALA		PS
Leelou Personal safety		PS
Life 360: Family Locator & GPS Tracker for Safety	PS	
Little Panda Travel Safety	PS	
Microsoft Family Safety	PS	
My Kids Safety—Family Tracker	PS	
My Safetipin: Complete Safety App	PS	PS
My SOS Family Emergency Alert	PS	PS
MySafeTravel	PS	
One Scream—personal safety	PS	PS
Panic Alarm	PS	
Panic Button — Anti-Theft, Emergency, Prank	PS	
Personal Panic Alarm	PS	
Personal Security & Travel Safety App—UrSafe	PS	
Power! Knowledge		VP
ProtectMe—Secure Video		PS
RAKSHA-Women Safety App	PS	
React Mobile—Safety App		PS
Rescue (2)—Personal Safety App	PS	
Rescuer: The Official Emergency Assistant	PS	
Safe Lagoon—Parental Control & Location Tracker	PS	
SafeNow App		PS
SafeON- Personal Safety App & Emergency Alert	PS	
Safety—Help—SOS	PS	
Safety App (Beta)	PS	
Safety App for Silent Beacon	PS	
Safety Light (Safety Light)—personal safety		PS
SEAM Personal Safety	PS	PS
Seecure®	PS	
Sekura	PS	
Shake2Safety—Personal Safety	PS	
Sister—Personal safety app	PS	
Smart Safe & Sound Panic app	PS	
SOS Alert | Emergency & Safety App	PS	
SOS Button—Family Locator for Safety and		PS
SOS fASTLANE		PS
StayVigil—Emergency Safety App	PS	
Stuck in a dark place		VP
The room beneath the rafters		VP
Track it EVEN if it is off | Antitheft SOS Family	PS	
TrackView	PS	
WanderSafe Beacon		PS
WanderSafe Safety App	PS	
WeeCare Health | Emergency Android App | Be Safe	PS	
WeHelp!—Personal Security	PS	
Woman Safety Resq	PS	
Women Safety	PS	

*PS* Personal safety, *VP* Violence prevention

^a^ Current app platform availability may differ to those shown

### Data analysis

App features were coded into two core functionalities: *incident assistance* (e.g., alarm systems, alert systems, evasive action, evidence capture) or *information generation and dissemination* (e.g., equipped users with knowledge to help recognise, manage, or prevent violence, contact details for service/support; see Table 2). As target user groups are widely used to inform the app design process [38], the target app user group was coded into: general population, women, families, lone individuals, and other vulnerable groups (see Table 3). Data were analysed in SPSS v.25. Descriptive statistics are used to detail the app features and functionality.

**Table 2:** App functionality definitions

Incident assistance	
Alarm systems	Designed to alert individuals in the user’s physical environment (e.g. flashing light, siren)
Evasive action	Enable users to evade or flee danger (e.g. ‘diversion calls’—which trigger a fake phone call; ‘duress pins’—fake pin codes which ostensibly unlock the phone, but send an emergency message)
Alert systems	Send electronic alert to a specified recipient(s) (e.g. pre-designated contacts)
Evidence capture	Allows the capture of violence through photos, audio and/or video. Some apps offer users the capacity to store this material remotely, in case damage occurs to their phone
**Information generation and dissemination**	
Monitoring others	Features which enable the monitoring of others including location tracking (e.g. show a user’s live location, or check-in on arrival at a predetermined location), geofence alerts (sent when a contact departs from a larger, pre-set boundary) and parental controls that allow monitoring and/or restriction of apps use
Educational materials	Materials containing violence prevention advice (i.e. recognising, tackling and preventing violence) including information on ongoing violent incidents (‘incident mapping’), and rates of violence in a given area. They also include skills-based approaches for violence mitigation and first-aid (e.g. self-defence tips). Apps can also include service/support contact information

**Table 3:** Overview of included applications

	**n**	**%**
**App user group**		
General population	45	52.3
Women	14	16.3
Families	11	12.8
Other vulnerable groups^a^	10	11.6
Lone individuals^b^	6	7.0
**App access requirements**^c^		
Location	74	86.0
User contacts	53	61.6
Microphone	36	41.9
Camera	39	45.3
Telephone	35	40.7
Messaging/SMS	21	24.4
Media	6	7.0
Bluetooth	14	16.3
Other^d^	8	9.3
**Connectivity**		
Requires 3G + or Wi-Fi to operate	67	77.9
**Cost**		
Free	52	60.5
Free, paid premium available	34	39.5
Optional paired device	11	12.8
**User rating (*n* = 62)**^**e**^		
1—< 4	18	29.0
4—5	44	71.0
Mean	4.0	

^a^ Including victims of domestic abuse, visually impaired, elderly and young people and those living with HIV/AIDS

^b^ Those living, working or travelling alone

^c^ App requested permission to grant access to these items on initial download and use

^d^ Calendar, notifications, movement/motion or light/torch

^e^ 24 apps had no user rating

Sixty-one apps had user reviews available. All available user reviews were extracted except those for one app which had in excess of 1 million reviews, to prevent bias towards a single app in sentiment analysis. Across the remaining 60 apps, the mean number of reviews was 206.83 (range 1–5,234). Thus, across all apps with more than 207 reviews, a random sample of 207 reviews were selected for analysis. A random sample of 207 reviews during the period of data collection were then extracted for the remaining app, resulting in a final sample for analysis of 3,820 app reviews. Extracted user reviews were coded by sentiment (negative, neutral, positive) by two reviewers (NJ and NG), with a third reviewer used to settle disagreement (KF). There was an excellent level of agreement in coding between reviewers (96.0%), Cohen’s κ 0.929. User reviews were analysed thematically using NVIVO. KF produced initial codes from the data, which were discussed with NG who also coded the data. Emerging themes were reviewed and agreed between researchers following an inductive approach. Researchers agreed the included quotes based on ensuring that they accurately represented the data and were comprehensive.

## Results

### App functions

Of the 86 apps, 52 (60.5%; Table 3) offered all functionality free of charge—the remaining apps offered additional features at a paid premium charge (one-off payment or subscription). 12.8% of apps could be used with an optional paired device (requiring additional purchase). Just over half (52.3%) targeted the general population, with 16.3% targeting women, 12.8% targeting families and 11.6% targeting other vulnerable groups. Upon download or first use, the majority of apps requested access to location services (86.0%), user contacts (61.6%), and under half requested access to the phone’s camera (45.3%), microphone (41.9%), or telephone (40.7%) respectively. Only 7.0% of apps requested access to other media (e.g., pictures). Most (77.9%) apps required a minimum of 3G or a Wi-Fi connection to operate.

Table 4 details the features of included apps. Seventy-two apps offered incident assistance and 56 offered information generation and dissemination (for definitions see Table 2). A fifth of apps (22.1%) offered an alarm system to actively help users be identified as in potential danger, of which all offered a siren, and 10.5% offered a flashing light facility. Fourteen apps (16.3%) offered evasive action, of which over half (57.1%) offered a duress pin, and 42.9% offered a diversion call facility. Of apps offering alert systems (70.9%), the majority were activated by button press (*n* = 53; of those with button press 90.6% in-app, 32.1% external button), although other methods of activation included location/movement sensor, pre-set timer, voice-controlled or through a paired device (see Table 4). All apps with alert systems sent alerts to designated contacts, with 16.4% offering to alert an emergency services operator (e.g., 999). However, the method of sending alerts to the recipient differed across apps, with the majority sending alerts through SMS (70.5% of apps with alert systems) and around one fifth used call (21.3%) or email (18.0%) methods, respectively. The majority (82.0%) of apps with alert systems included the user location within the alert. Just over a third (33.7%) of all apps offered evidence capture, of which the majority featured photo (75.9%) or video (62.1%) capture. Remote storage for evidence was offered by 11.6% of apps.

**Table 4:** App features and functionality

	**N**	**% apps with attribute**	**% of all apps**
***Incident assistance***	***72***		***83.7***
**Alarm systems**	**19**		**22.1**
Siren	19	100.0	22.1
Flashing light	2	10.5	2.3
**Evasive action**	**14**		**16.3**
Diversion Call^a^	6	42.9	7.0
Duress pin^b^	8	57.1	9.3
**Alert systems**	**61**		**70.9**
Alert activation			
Button press	53	86.9	61.6
(in app)	48	78.7	55.8
(external button)	17	27.9	19.8
Location/movement sensor^c^	9	14.8	10.5
Paired device	10	16.4	11.6
Pre-Set timer	8	13.1	9.3
Voice controlled	5	8.2	5.8
Alert recipient(s)			
Designated contact(s)	61	100.0	70.9
Emergency service operator	10	16.4	11.6
Nearby app users	1	1.6	1.2
Method of sending alert to recipient(s)^d^			
SMS	43	70.5	50.0
Call	13	21.3	15.1
Email	11	18.0	12.8
Social media	3	4.9	3.5
In app notification (to other app users)	11	18.0	12.8
Alert content			
Location	50	82.0	58.1
Safety check-in	16	26.2	18.6
Other^e^	4	6.6	4.7
**Evidence capture**	**29**		**33.7**
Photo capture	22	75.9	25.6
Video recording	18	62.1	20.9
Audio recording	11	37.9	12.8
Other^f^	4	13.8	4.7
Remote Storage	10	34.5	11.6
***Information generation and dissemination***	***56***		***65.1***
**Monitoring others**	**45**		**52.3**
Location tracking	36	80.0	41.9
Geofence alert	5	11.1	5.8
Check-in (location)	20	44.4	23.3
Parental control^g^	3	6.7	3.5
**Educational Information**	**22**		**25.6**
Service/support contact information	5	22.7	5.8
Violence prevention advice^h^	14	63.6	16.3
Violence mitigation and first aid	3	13.6	3.5
Game play	4	18.2	4.7

^a^ Fake call to the user’s phone

^b^ Fake pin code sends an alert

^c^ Including accident/fall detection

^d^ Some apps use multi-methods

^e^ SOS message without location, accident/fall alert, live video stream

^f^ Storage of other evidence including messages, emails, and social media

^g^ App blocker/monitor, screen time limit

^h^ Incident mapping, safety audit, recognising signs of violence

Over half (52.3%) of included apps enabled the user to monitor other app users, predominantly through location tracking or a check-in facility (Table 4). Parental control was offered by 6.7% of the apps categorised as enabling the monitoring of others. A quarter of apps offered educational information (Table 4).

### User rating and reviews

Of the sixty-two apps with a user rating, 71.0% (*n* = 44) received a user rating of 4–5 stars (mean 4.0). There was no association between user rating and app features and functionality (see Additional Table 1). Over half (52.4%) of user reviews were rated as having positive sentiment, while 38.8% were negative and 8.8% were neutral. Additional thematic analyses resulted in the identification of the themes: (1) positive consequences of app use; (2) technical and usage issues; (3) financial dissatisfaction; (4) personal data and ethical issues.

#### Positive consequences of app use

User reviews indicated that apps were easy to use and cited a number of positive consequences of app use, including being able to stay connected with friends and family. Several reviews indicated that apps provided users with increased reassurance, confidence and perceptions of security. Frequently, *‘peace of mind’* was cited as a consequence of app use:

“I really like this app and it gives me peace of mind when staying alone or walking by myself”

“It’s the app I never hope to use, but I am so glad it’s here. It gives me peace of mind if I’m ever walking home at night and feeling uncomfortable - something that happens a lot in [PLACE]”

“Love this app, gives me peace of mind since it allows me to allocate my loved ones at any time”

The use of intelligent and unobtrusive technology were highlighted as positive app features, with location tracking particularly valued. Some reviews indicated an intention to recommend apps to their wider social network, especially to vulnerable user groups, for example, lone females and children:

“We need more platforms like these to make us more aware on safety issues in the public sphere. Loved the initiative”

“Very useful app ..Must install for all Women irresepctive *[sic]* age”

“I’ll be recommending it to lone worker colleagues & friends”

#### Technical and usage issues

The most common theme across all user reviews was technical and usage issues, including problems with app download, registration, and app functionality following software updates. Technical issues were a source of great frustration for many users:

“The concept is good but none of the features worked!”

“App keeps crashing every time”

Importantly, a large number of reviews highlighted concerns over app reliability and questioned the ability of the app to function in the event of an emergency and meet its stated aim to protect the user and provide security. Consequently, some users considered apps with impaired operability potentially unsafe and unusable:

“I just can’t help but thinking that somebody could get hurt relying on this app to keep them safe”

“This app sent out an SOS to my contacts when I hadn't triggered it... I had a lot of distressed calls afterwards”

#### Financial dissatisfaction

A large number of reviews highlighted user dissatisfaction with the financial cost of some apps. Including user reports of poor clarity on the economic requirements of some apps before download and also where apps had been freely available, but later required financial payment/subscription for additional features:

“Isn't free at all, you have to pay to use literally any of the features.”

“When the app states "one month unlimited use" be aware it is indeed a monthly subscription renewed automatically and undefinable”

Furthermore, some users stated they had difficulty cancelling app subscriptions, with continued payment being taken for apps they no longer used.

#### Personal data and ethical issues

A number of reviews raised concerns over the personal data collected by apps, particularly where apps required unexplained access to personal information which seemed irrelevant to the user, such as photos, contacts, or details of social media accounts:

“Wants access to all my files and photos, no way, that's irrelevant and an invasion of my privacy. Why does it need my files and photos?”

“I was really interested in this app- however having started to sign up and read the terms I was worried to read that my personal info- name, social media, email etc could be shared with unnamed 3rd parties”

A number of individuals also queried the ethics of apps designed to provide personal safety and violence prevention, feeling that app developers were taking advantage of vulnerable individuals. Further to this, some reviews highlighted the misuse of app features (e.g., location tracking) with users indicating that they felt that their privacy had been breached, where individuals had used the apps to control their movements without their knowledge or consent:

“I find it pretty awful and disgusting that pretty much everything on this app is a premium feature. That people will build this to profit off people's fear and have a pay wall for feeling safe when there are so many cases of assault”

“A disgusting invasion of privacy”

## Discussion

A large number of freely available apps designed to enhance personal safety and prevent interpersonal violence were identified. Evidence from user ratings and reviews of the apps studied indicated generally a positive response towards the apps. However, reviews also highlighted a number of recurrent usage issues, user dissatisfaction with clarity on pricing and concerns over app ethics and privacy. Whilst previous research has considered the potential impact of technology on public health [39, 40], this study increases our understanding of the nature of smartphone apps in the UK designed to promote a user’s safety. To our knowledge, it is the first study to provide a broad overview of smartphone applications to prevent interpersonal violence in the UK which includes an examination of the app user experience. The findings presented here are of relevance to app designers and developers, regulators and a wide range of organisations who seek to implement interventions for violence prevention, including policymakers. Whilst the review focused on apps available in the UK, the findings are likely to reflect issues which may be present in many other high-income countries and, therefore, may have implications for app development in other countries.

The volume of app reviews identified in this study indicates that smartphone apps appear to be a popular and desirable resource for violence prevention. For the apps reviewed in this study, the majority had a positive user rating and over half of app user reviews had a positive sentiment. User reviews indicated perceived app usefulness and a regular reliance on such apps for their own safety and that of friends and family. A range of positive consequences of app use were identified across user reviews, including apps meeting their stated aim of increasing user feelings of safety, providing ‘peace of mind’ and improving user confidence. Although positive outcomes, it is important to acknowledge that increased perceptions of safety or confidence as a result of app ownership may lead users to engage in situations that actually pose an increased risk to their personal safety. Research has identified that individuals who feel safer as a result of having a mobile phone are more likely to engage in risky behaviour [13]. It is therefore important for further research to establish if increased confidence or feelings of safety associated with app ownership influences the extent to which individuals enter potentially risky situations or areas. Furthermore, the findings here indicate a lack of evidence on the effectiveness of apps for violence prevention, thus further evaluation of such apps is required.

Despite the majority of apps receiving high user ratings, the most common theme identified across user reviews was that of technological issues, whereby apps did not work upon download or registration or operated inconsistently. Reviews also commonly highlighted issues with app reliability, impacting on app use and the ability for apps to meet their stated aim – to protect users. Some reviews also indicated that running apps in the ‘background’ of their smartphone led to battery depletion, leading to the risk for no available smartphone communication features if they were required. Research methodologies to examine apps are only generally in the early stages of development, with prior investigations focusing on smartphone app use in relation to lifestyle-associated behaviour change such as smoking cessation and weight management [41, 42], physical activity [43] and the management of chronic health conditions (e.g., hypertension, heart disease and diabetes [44–46]). It is imperative to understand both the benefits and potential caveats of apps designed and marketed to reduce and prevent interpersonal violence. Findings here show that there is little or no evidence base for such apps. However, there is a clear need for the formal evaluation of commercially available smartphone apps, a technology that individuals may rely on in life-threatening situations.

UK Government has indicated the potential usefulness of apps for personal safety and violence prevention [23], and in 2021, a number of Police Forces in the UK urged individuals to download and use a specific smartphone app to help maintain their safety (for an example, see [47]). Furthermore, in the same year, UK media highlighted the usefulness of apps to prevent violence in response to a number of female homicides. However, such apps are currently unregulated and not tested to any standards. It is essential that apps available for use are secure and work properly and that the information they provide is accurate and evidence-based, especially when relied upon for individuals' safety. It is important that time is taken to consider if regulation or a measure of app quality is needed. Legislation may be required for the development and testing of apps marketed for violence prevention in order to reduce the availability of apps that are unreliable and, therefore, potentially dangerous. Such work would require input from expert groups with representation from public sector and criminal justice organisations, health, academia and violence prevention groups. With such public recommendations for app use, including from UK Government and criminal justice agencies, it is imperative that the outcomes associated with app use are fully understood, otherwise the promotion of such apps risks vulnerable people relying on largely untested technology in potentially dangerous circumstances.

Critically, it is essential to understand the efficacy of apps marketed for violence prevention in real-life incidents of interpersonal violence. Although apps may support incident assistance and the collection of evidence, they do not necessarily prevent violence from occurring. Effective strategies for violence prevention include education, changing cultural norms that support violence and promoting gender equality [48]. Such information could be provided through an app platform, yet very few of the apps identified here offered educational information to influence social norms against violence, advice on how to mitigate violence or recognise the signs of violence or incident mapping to enable users to avoid areas with high levels of violence or ongoing incidents. The impacts of the provision of such information and facilities are unclear. It was evident from user reviews that many users recommended the apps to others. However, this does not suggest changing attitudes towards violence, more that individuals feel a need to encourage others to protect themselves against it. Campaigners against the promotion of policy encouraging the use of apps to prevent violence also highlight that apps do not address the root causes of such violence [23].

Findings here, raise questions over the ethics of the development of apps designed to prevent violence, indicating public perceptions that such apps target vulnerable individuals and should not charge for functions that would offer protection. Despite the majority of identified apps targeting generic users, over one in ten were marketed specifically for women, families or other vulnerable groups. In a sample of female Italian University students, only a third of participants were aware of the existence of apps to assist women experiencing violence, and awareness was lower amongst women with little knowledge of the prevalence of violence against women [49]. Personal safety apps are most likely to attract users from already vulnerable groups and those who are already security conscious [49, 50]. However, apps may not be accessible to those most in need if such individuals have difficulty using the technology or are unable to afford the technology required to operate such apps. User reviews also highlighted dissatisfaction over the clarity of app pricing. While some reviews in this sample considered the financial costs of subscription apps as unfair, a large number of available free applications to prevent violence were identified. Given that those of low socioeconomic status are at increased risk of interpersonal violence [51], it is important that such apps, where they are effective, are freely available.

With unnecessary access requirements to personal data noted as a concern in some reviews, app developers should provide further clarity to potential users on why access to data items is required for app use [52, 53]. Where necessary, developers may wish to provide users with the flexibility to accept a minimum level of data sharing requirements thus, minimising potentially unnecessary data collection. Processes such as these may support a sense of control amongst app users, potentially given that app users already are taking steps to improve their personal safety. It should be noted that despite location tracking features specifically valued by a number of users, a range of smartphones now offer the ability to share a user’s location as standard (although these are not marketed by mobile phone developers to improve personal safety). Apps for violence prevention commonly utilise mobile phone features which are usually standard components of smartphone design (e.g. camera, light, location sharing). The requirement of apps therefore is potentially mitigated, if users are able to understand how they can apply these common features to themselves [54, 55]; for example, share their location with a family member or set up in case of emergency contacts (ICE) – features commonly available on smartphones. A study amongst college women in the USA identified that a primary reason for not using an app designed to reduce sexual victimisation risk was that the app was redundant due to the provision of generic features smartphones that were easier to use [56].

The majority of apps offered incident assistance such as alert systems. However, the majority of alert systems were activated in-app. This method of operation places reliance on a user’s capability to access and operate apps easily and quickly in the event of violence – a process that may be critically inefficient or impossible when faced with a dangerous situation. Functionality, usability and performance are rated as the most valuable features of mobile technology [57]. For apps to work effectively, users need to fully understand how apps operate and their limitations. As such, app developers should look to provide users with comprehensive instructional tutorials, and requirements for users to test their apps after download to ensure the app is working correctly and users fully understand its functionality.

Our findings indicate a need for further research on smartphone apps for violence prevention. Such work should also seek to identify any potential unintended consequences associated with app use. For example, apps that identify ‘unsafe’ locations could result in decreased footfall in such areas, which may exacerbate trends for violent crime, according to the principle of ‘eyes on the street’ [58], which poses that an active social presence may deter criminal activity due to the potential of witnesses. Research may also try to understand other ways in which such apps may reduce levels of violence, for example, the widespread use of apps could influence criminals’ perception of the likelihood that they will be identified and prosecuted.

There are a number of study limitations which should be acknowledged. Due to the lack of established frameworks to evaluate apps marketed for violence prevention, we utilised mixed methods to explore app utility and use. The sample was limited to English language apps currently freely available in the UK, thus work is required to extrapolate these findings to other geographical contexts. Other relevant apps may be available that were not identified in our searches. However, we searched the two most common application stores using systematic and replicable terms. In line with other studies assessing the use of apps for violence prevention [59], only freely available apps and app features which were understood to be freely available at the time of data collection were reviewed, thus apps or features/functions within apps which required a financial cost are not reviewed here. This may be a source of bias, as it is possible that paid apps may have had more testing and could potentially be more reliable or have been subject to evaluation. However, free apps or those with free basic functionality are likely to attract attention from the general public and potentially have higher rates of use. Future work should consider exploring such offerings and comparing the difference between free and paid offerings. We were unable to access data on the number of individuals who use these apps and how regularly such apps are used; such information would be key to better understanding how widespread app use is within the UK. It should also be noted that there might be a substantial number of app users who have not provided an app review or numerical rating and we are unable to identify any bias this may have caused. Only a subset of apps had available user ratings (*n* = 62). Furthermore, although a large number of online user reviews were analysed, these related to 70.1% of the included apps (61/86) and to prevent bias towards a single app in sentiment analysis, we were unable to include all app reviews for apps which had substantially large numbers of user reviews. As such, the qualitative findings are not fully representative of all included apps and user perceptions of them and may be biased as the apps with the most reviews may be the most commonly used. Furthermore, included user reviews may have related to previous app versions, therefore any issues identified may have been resolved by the app developer in recent app updates. However, we included user reviews over a three-year period to capture a range of issues and common themes were identified across a large number of reviews. Excluding app reviews not in English language may have biased the findings and potentially resulted in those for whom English is not their first language being underrepresented. However, app versions across regions may have varied and our focus remained on apps available in the UK. Finally, other apps may offer features that may promote personal safety (e.g. location sharing), which are not included here. However, the focus of the manuscript was to identify apps marketed to users as useful for personal safety and or preventing violence.

## Conclusions

A range of smartphone apps marketed to prevent violence and improve personal safety are freely available in the UK. However, the role of such apps in reducing the devastating and costly impact of interpersonal violence is unclear. Findings here indicate that many individuals report finding such apps useful and consequently may place a reliance on them for their safety. Despite apps claiming to enhance personal safety by offering quick and easy communication, location tracking, alarm services and evidence recording, user reviews indicated major concerns over the unintended harms of apps, poor reliability and their potential for misuse. Learning identified here includes the need for app developers to be transparent as to the financial cost to users of apps as well as their limitations and personal data requirements. Already in the UK, Government has highlighted the potential of apps as both a preventative measure and response to violence, particularly for violence against women. Our findings have implications for such policy or discussions as they highlight a need to further evaluate apps for personal safety and to consider if regulation or a measure of app quality is required. Whilst apps to promote behaviour change are often subject to meticulous evidence-based testing, the same principles are not necessarily applied to apps designed for personal safety despite potentially vulnerable individuals relying on them for their protection. Scientific evidence does not currently back up claims from app developers used to market such apps, that they are effective for improving personal safety. User reviews here highlight both flaws in app design and reliability, indicating that some apps may cause harm by compromising the privacy of an individual’s location or other personal data. Therefore, it is concerning that individuals may rely on the use of unregulated apps for their personal safety. Further research on the use of apps for violence prevention is required which should investigate how tools to prevent violence through smartphone technology better demonstrate quality assurance, effectiveness and personal data security.

## Supplementary Information

**Additional file 1: Additional Table 1.** App features and functionality by user rating.

## Data Availability

The datasets analysed during the current study are available from the corresponding author on reasonable request.

## Abbreviations

App: Application
GPS: Global Positioning System
ICE: In Case of Emergency
UK: United Kingdom
VAW: Violence against women
