# Supplementary material for: The use of mobile phone applications to enhance personal safety from interpersonal violence – an overview of available smartphone applications in the United Kingdom
Source: BMC Public Health. 2022 Jun 9;22:1158. doi: 10.1186/s12889-022-13551-9 (PMC9185885; doi:10.1186/s12889-022-13551-9)
Supplement: Supplementary file 1 — Additional file 1: Additional Table 1. App features and functionality by user rating. [file 12889_2022_13551_MOESM1_ESM.docx]

Additional Table 1: App features and functionality by user rating

|  | **% User rating** | | ***X*^2^** | **P** |
| --- | --- | --- | --- | --- |
|  | **1 - < 4** | **4 - 5** |  |  |
| ***Incident assistance*** |  |  |  |  |
| Yes | 25.9 | 74.1 |  |  |
| No | 50.0 | 50.0 | 1.960 | 0.162 |
| **Alarm systems** |  |  |  |  |
| Yes | 25.0 | 75.0 |  |  |
| No | 30.4 | 69.6 | 0.170 | 0.680 |
| **Evasive action** |  |  |  |  |
| Yes | 33.3 | 66.7 |  |  |
| No | 28.0 | 72.0 | 0.134 | 0.715 |
| **Alert systems** |  |  |  |  |
| Yes | 26.7 | 73.3 |  |  |
| No | 35.3 | 64.7 | 0.446 | 0.504 |
| **Evidence capture** |  |  |  |  |
| Yes | 22.7 | 77.3 |  |  |
| No | 32.5 | 67.5 | 0.658 | 0.417 |
| ***Information generation and dissemination*** |  |  |  |  |
| Yes | 32.4 | 67.6 |  |  |
| No | 24.0 | 76.0 | 0.515 | 0.473 |
| **Monitoring others** |  |  |  |  |
| Yes | 37.5 | 62.5 |  |  |
| No | 20.0 | 80.0 | 2.302 | 0.129 |
| **Educational Information** |  |  |  |  |
| Yes | 14.3 | 85.7 |  |  |
| No | 33.3 | 66.7 | 1.909 | 0.167 |
